# Purslane-Fortified Yogurt: In-Line Process Control by FT-NIR Spectroscopy and Storage Monitoring

**DOI:** 10.3390/foods14122053

**Published:** 2025-06-11

**Authors:** Ayse Burcu Aktas, Silvia Grassi, Claudia Picozzi, Cristina Alamprese

**Affiliations:** 1 Department of Biochemistry, Faculty of Science, Sivas Cumhuriyet University, 58140 Sivas, Turkey; burcuaktas@cumhuriyet.edu.tr; 2 Department of Food, Environmental and Nutritional Sciences (DeFENS), Università degli Studi di Milano, 20133 Milan, Italy; silvia.grassi@unimi.it (S.G.); claudia.picozzi@unimi.it (C.P.)

**Keywords:** yogurt fortification, lactic acid fermentation, rheology, Gompertz, texture

## Abstract

Yogurt fortification with purslane (*Portulaca oleracea* L.) can improve its health benefits, but it may alter the fermentation step and its final properties. Thus, the current study investigated the suitability of Fourier Transform-Near Infrared (FT-NIR) spectroscopy for in-line monitoring of lactic acid fermentation of purslane-fortified yogurt compared with fundamental rheology. Changes in the yogurt properties during storage were also assessed. Set-type yogurts without and with lyophilized purslane leaves (0.55%) were produced and stored at 4 °C for up to 18 days. Lactic acid bacteria concentrations before and after fermentation at 43 °C for 2.5 h showed that the presence of purslane did not interfere with bacterial growth. The purslane addition increased the milk viscosity, resulting in a yogurt with complex modulus values higher than those of the reference sample (360 vs. 172 Pa). The elaboration of spectral data with Principal Component Analysis and the Gompertz equation enabled calculation of the kinetic critical points. Applying the Gompertz equation to the rheological data, it was evident that FT-NIR spectroscopy detected earlier the fermentation progression (the critical times were about 18% earlier on average), thus enabling better control of yogurt production. No significant changes in microbial or textural properties were noted during yogurt storage, demonstrating that purslane addition did not affect the product stability.

## 1. Introduction

Yogurt is a dairy product with multiple processing steps, including milk standardization, homogenization, heat treatment, and fermentation [1]. Milk gelation during fermentation is a physicochemical transition in which milk transforms from a low-viscosity Newtonian fluid into a semi-solid or solid gel-like matrix [2]. Gelation is induced by lactic acid bacteria, which progressively acidify the milk, leading to structural modifications of casein and the formation of a porous protein network that entraps the serum phase. The denatured whey proteins also contribute to the density of the protein network and change the gel properties depending on the initial heat treatment of the milk [3,4]. Recent studies have indicated that the sequence of yogurt production steps, processing temperatures, and shear intensity significantly influence the characteristics of the final product during storage [5,6]. The changes in gel characteristics of yogurt caused by the modifications in formulation or production technique are typically connected with changes in microstructure. The differences in network crosslinking density, pore size, network heterogeneity, and protein particle size all have an impact on gel properties [4].

Fortified yogurts are described as yogurts that have been supplemented with nutrients or other types of ingredients [7]. Fortification of yogurts enables the development of functional foods beneficial to human health [8]. Previous works evaluated the effects of yogurt fortification with different plant-derived compounds, as reported in the recent review by Wazzan [9]. In particular, there has been a growing interest in using *Portulaca oleracea* L., also known as purslane, as a new ingredient [10,11,12,13]. Purslane is commonly cultivated and consumed in Italy, Turkey, Spain, Greece, the UK, Iran, Malaysia, the Philippines, China, North Africa, Australia, the USA, Brazil, and Mexico [14]. It has numerous nutritional, therapeutic, pharmaceutical, and phytoremediation benefits. Purslane could be a good plant ingredient for yogurt fortification due to its high nutrient content, low resource requirements for cultivation, and adaptability to different environmental conditions [15]. The stems and leaves of purslane are edible and contain nutrients such as omega-3 fatty acids, vitamins A, C, and E, electrolytes like potassium and sodium, and minerals like calcium, magnesium, and phosphorus [16]. Purslane contains considerable amounts of alpha-linolenic acid, which could be associated with cardiovascular health benefits, making it an important plant source for human nutrition to be added in functional foods [17]. Purslane also contains a variety of phenolic compounds (e.g., gallic, protocatechuic, p-hydroxybenzoic, chlorogenic, vanillic, caffeic, syringic, and p-coumaric acids), as well as flavonoids (e.g., rutin, myricetin, quercetin, apigenin, kaempferol, isoquercetin, luteolin, genistein, and ferulic) [18,19].

As fortification can affect yogurt fermentation and the properties of the final product, the implementation of automated monitoring and process control systems may significantly benefit the dairy industry by enhancing product quality and generating economic advantages. Different approaches have been explored and proposed for continuous lactic acid fermentation monitoring [20,21,22,23,24], among which the ones employing near-infrared (NIR) spectroscopy have high potential [25,26,27,28]. In particular, NIR and Fourier Transform (FT)-NIR spectroscopy proved able to provide significant real-time information compatible with rheological and conventional quality parameters of yogurt [26,28]. Similarly, NIR spectroscopy applied to the monitoring of lactic acid fermentation of Bulgarian yogurt provided results that considerably exceeded those obtained by monitoring only the pH, which is a simple process parameter of yogurt fermentation [29].

To the best of our knowledge, no studies have been published so far on the use of FT-NIR spectroscopy to monitor fermentation of purslane-fortified yogurts. Therefore, this study evaluated the applicability of FT-NIR spectroscopy, in combination with multivariate data analysis, for monitoring lactic acid fermentation in purslane-fortified set-type yogurts and compared its performance with that of fundamental rheology. In addition, the microbiological and textural properties of the yogurts during storage were assessed. The ultimate goal is to offer yogurt manufacturers new insights into product development and process control, enabling them to meet consumer expectations and enhance market competitiveness.

## 2. Materials and Methods

### 2.1. Yogurt Ingredients

Ultra-high temperature processed (UHT) milk was used for yogurt production (Latteria Soresina, Milan, Italy). UHT milk was chosen primarily for its extended shelf life and microbial stability, which enabled a better control of the experimental conditions, while minimizing variability due to background microflora. Moreover, the use of UHT milk helped in creating environmental conditions more similar to those of industrial yogurt production, where pasteurization is performed under time-temperature conditions stronger than those applied for milk to be sold as a pasteurized product. The milk fat content was 3.6%, and the dry matter of the milk was standardized to 17.9% by adding skim milk powder (Dolcincasa, Reggio Emilia, Italy) to obtain a harder gel after fermentation [30]. The starter culture used (Y551, 10 U; Maysa Gida, Istanbul, Turkey) contained *Lactobacillus delbrueckii* subsp. *bulgaricus* and *Streptococcus thermophilus*. The purslane plant was purchased in a local market (Sivas, Turkey) and its leaves were frozen overnight at −80 °C prior to lyophilization, carried out at a pressure of 10^−3^ mbar, for 4 days in a freeze-drier (Labconco, Kansas City, MO, USA). The lyophilized purslane leaves were then powdered in a chopper (Kenwood Ltd., Havant, UK) and employed in the yogurt production.

### 2.2. Yogurt Production and Storage

The laboratory-scale production of purslane-fortified set-type yogurt (PFY) was performed in duplicate according to the procedure published by Satkin et al. [10], adopting the optimized concentrations of lyophilized purslane and starter culture. For each fermentation trial, 850 mL of standardized milk supplemented with 0.55% lyophilized purslane leaves was inoculated with 3.41% starter culture and divided into 50 mL sterile polypropylene screw cap containers. The lactic acid fermentation was carried out in an incubator (UFE500 Memmert GmbH, Schwabach, Germany) at 43 °C for 2.5 h, until reaching a pH of 4.6 ± 0.2 evaluated by means of a pHmeter SevenEasy (Mettler Toledo, Columbus, OH, USA) previously calibrated with pH 4 and 7 buffers. A standard yogurt (without purslane addition) was also produced in duplicate as a reference (REF), using the same procedure.

At the end of fermentation, the yogurt samples were stored in a cold chamber (4 ± 2 °C) for 5, 12, and 18 days.

### 2.3. Monitoring of Fermentation

Both PFY and REF samples were continuously monitored during fermentation by means of FT-NIR spectroscopy and rheological measurements, according to the procedures by Grassi et al. [28].

Immediately after the starter inoculation, a 100 mL aliquot of sample contained in a glass bottle was placed in a thermostatic bath at 43 ± 1 °C for 2.5 h. The fiber-optic probe of a MPA FT-NIR spectrometer (Bruker Optics, Milan, Italy) equipped with a transflector (1 mm pathlength) was inserted directly in the sample, then covered with aluminum foil to avoid sample evaporation. The spectra were collected every 15 min in the 12,500–4000 cm^−1^ range, with a resolution of 16 cm^−1^, and 64 scans for background and sample. OPUS software (v. 6.5, Bruker Optics, Milan, Italy) was used to control the instrument and collect the data.

Rheological measurements were performed on inoculated samples (19 mL) by using a Physica MCR 102 rheometer (Anton Paar GmbH, Graz, Austria) managed by the software RheoCompass (v. 1.21, Anton Paar GmbH, Graz, Austria). A dynamic oscillatory test was carried out at 43.0 ± 0.1 °C using concentric cylinders (CC27) with constant 1% strain and 1 Hz frequency. A solvent trap containing deionized water was used to avoid sample evaporation during the test. The complex modulus (G*) was measured every 2 min for the whole fermentation process (2.5 h). At the end of the test, the resulting gel was evaluated for viscoelastic properties by means of frequency sweep (frequency range 0.1–1 Hz; strain 0.01%) and strain sweep (strain range 0.001–10%; frequency 1 Hz) tests.

### 2.4. Microbiological Analyses

Lyophilized purslane leaves were subjected to a preliminary analysis for aerobic mesophilic microorganisms according to ISO 4833-2:2022 [31]. Microbiological analyses on yogurt were performed in duplicate both on REF and PFY samples before and after fermentation, as well as after 5, 12, and 18 days of storage at 4 ± 2 °C. Samples (10 g) were aseptically collected and resuspended in a 20 g/L sodium citrate solution (Sigma–Aldrich, St. Louis, MO, USA) to obtain the first decimal dilution. After homogenization, appropriated serial dilutions in physiological solution (9 g/L NaCl, pH 7) were made in duplicate and plated on De Man–Rogosa–Sharpe (MRS) agar (Merck KGaA, Darmstadt, Germany) for counting *Lb. bulgaricus* and on M17 agar (Merck KGaA, Darmstadt, Germany) for *Str. thermophilus*. Incubation was performed at 37 °C for 48 h in anaerobiosis jars for the MRS plates. Moreover, appropriate dilutions were plated on yeast extract glucose chloramphenicol (YGC) agar (Liofilchem s.r.l., Roseto degli Abruzzi, Italy) and incubated at 25 °C for 48 h to evaluate the possible presence of yeast and molds as indicators of purslane or environmental contamination. Results are reported as the weighted average (with standard deviation) values of the log-number of colony forming units per gram of sample (log CFU/g) obtained for the two production replicates of each yogurt type.

### 2.5. Texture Analysis

At the end of fermentation (after 2 h cooling in a cold room at 4 ± 2 °C) and during storage at 4 ± 2 °C up to 18 days, REF and PFY samples (50 g) were analyzed in triplicate for mechanical properties using a 3365 Instron Universal Testing Machine managed by the software Bluehill v. 2.9 (ITW Test and Measurement Italia S.r.l., Pianezza, Italy). A penetration test (15 mm final penetration) was performed on cold samples at a constant rate of 50 mm/min, with a 36 mm diameter plate connected to a 100 N load cell [32]. Results are expressed as the average and standard deviation values of the firmness obtained in the two production trials of each yogurt type, calculating the load (N) at 10 mm penetration.

### 2.6. Data Processing

Results of microbiological and textural analyses were analyzed by one-way analysis of variance (ANOVA) followed by the Least Significant Difference test (LSD) to detect possible significant differences between samples (*p* < 0.05). ANOVA was performed with Statgraphics Centurion software (v. 18.1.13; Statistical Graphics Corp., Herndon, VA, USA).

FT-NIR spectra were reduced in the range 8900–5555 cm^−1^ to eliminate noisy and not informative regions. Then, they were pre-processed by smoothing (moving average, 11-point segment size) and Standard Normal Variate (SNV), and transformed into the first derivative (Savitzky-Golay algorithm, polynomial order = 2, gap size = 11 data points) to minimize the effect of baseline shifts. The pre-processed spectra were mean-centered and explored by means of Principal Component Analysis (PCA) using The Unscrambler software (v. 9.8, Camo Software AS, Oslo, Norway). The PC1 scores of each fermentation trial were normalized from 0 to 1 and described as a function of fermentation time by the Gompertz equation implemented in JMP Pro software (v. 17.2.0, JMP Statistical Discovery LLC, Cary, NC, USA). The same equation was used to describe the logarithmic complex modulus values of each fermentation trial. The first (d1) and the second (d2) derivatives of the curves were then calculated to identify kinetic critical points (i.e., maximum rate, acceleration and deceleration times) [28].

## 3. Results and Discussion

### 3.1. Fermentation Process Control

Milk fermentation was monitored by evaluating the concentration of microbial starter before and after incubation, as well as measuring in real-time the FT-NIR spectra and the rheological properties. Table 1 shows the concentrations of the lactic acid bacteria in the two types of samples (i.e., without and with the lyophilized purslane addition) before and after fermentation at 43 °C for 2.5 h. Due to the starter composition, a higher concentration of *Str. thermophilus* rather than *Lb. bulgaricus* was found in both samples at the beginning and the end of the fermentation, although the difference was significant (*p* < 0.05) only for the REF sample after 2.5 h. No significant differences (*p* > 0.05) in the concentration of each strain were observed between the two evaluated time points, likely due to the high inoculum concentration, or between the two yogurt types. The pH of the samples at the end of fermentation was in the range 4.6–4.8, indicating the correct acidification of all of the produced yogurts [30] and highlighting that the presence of the lyophilized purslane leaves did not interfere with lactic acid bacteria proliferation. Compared to conventional industrial production, acidification occurred more rapidly, enabling the completion of yogurt fermentation in 2.5 h instead of the typical 6–8 h. This acceleration could be attributed to the specific microbial starter culture employed and the high inoculum level.

Yeast and mold were always lower than 10 CFU/g, indicating that the addition of the lyophilized purslane leaves did not present critical issues for the safety of the final product. The count of microbial colonies on purslane leaves was deemed satisfactory, showing a value of 3.9 log CFU/g.

**Figure 1:**
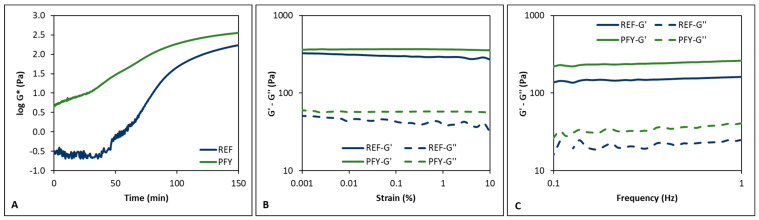
Average time sweep (**A**), strain sweep (**B**), and frequency sweep (**C**) curves registered for the reference (REF) and purslane-fortified yogurt (PFY). G*, complex modulus; G′, storage modulus; G″, loss modulus.

The textural properties of fermented milk gels are of paramount importance for yogurt quality and consumer acceptability [24]. For this reason, an on-line monitoring of the curd development through rheological analysis was set up. Figure 1A shows the average time sweep curves obtained for the two types of yogurts in terms of complex modulus (G*). It is well established that structure development of yogurt is related to the coagulation phenomena, including calcium caseinate–phosphate complexes destabilization, interactions between casein micelles and denatured whey protein, and casein coagulation [1]. These phenomena resulted in the typical sigmoid trend of the time sweep curves reported in Figure 1A. A sigmoid curve of apparent viscosity as a function of fermentation time was also reported in a previous study [24]. From Figure 1A, it is also evident that the presence of the lyophilized purslane leaves increased the viscosity of milk before fermentation, resulting in a final product with higher viscoelasticity properties compared to the REF sample (i.e., 360 vs. 172 Pa, complex modulus). This can be due to the higher total dry matter of milk fortified with dried purslane [12] and to the thickening effect of the high fiber content of purslane leaves, as reported in the literature (8% dried weight) [33]. Indeed, similar results were obtained from fortifying yogurt with apple pomace or carrot cell wall particles due to the presence of pectin, cellulose, and hemi-cellulose [8,34]. In another work, the addition of a purslane extract resulted in a yogurt with higher viscosity, which was attributed to the interaction between phenolic compounds and the milk proteins [13]. 

The higher firmness of the PFY was confirmed by the strain sweep and frequency sweep test results (Figure 1B,C), showing higher values of elastic (G′) and loss (G″) modulus for PFY compared to REF. In detail, in the strain sweep test, the average value of G′ and G″ was 367 and 57 Pa, respectively, for PFY compared to 303 and 43 Pa for REF. Similarly, the frequency sweep test revealed G′ and G″ average values of 241 and 34 Pa for PFY and 150 and 22 Pa for REF, respectively. Thus, the rheological analyses confirmed that the addition of lyophilized purslane leaves did not impair the development of the yogurt structure and even improved the strength of the final gel.

**Figure 2:**
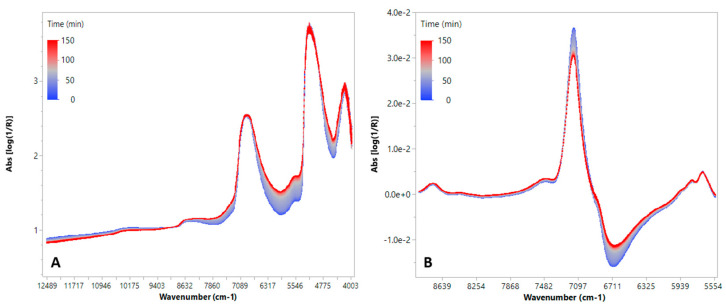
FT-NIR spectra collected during one of the fermentation trials: (**A**) raw spectra; (**B**) pre-processed spectra.

The efficacy of NIR spectroscopy for in-line monitoring of milk lactic acid fermentation was already demonstrated in previous works [26,27,28,29]. Thus, to check the reliability of FT-NIR spectroscopy as a simple, cheap and robust tool for the monitoring of yogurt production in the presence of new ingredients, the fermentation step was continuously monitored by acquiring FT-NIR transflectance spectra every 15 min for 2.5 h (Figure 2). The raw spectra showed a typical trend as a function of fermentation time (Figure 2A), with no relevant differences in spectral features or trends between PFY and REF. The trends observed in the spectra were similar to those reported in other works [26,28,35]. In particular, the FT-NIR spectra highlighted both scattering and absorption phenomena; scattering is primarily associated with textural changes, while absorption is related to biomass development and the conversion of sugars into lactic acid [26]. The dominant peaks at 6900 and 5100 cm^−1^, which are related to the O–H first overtone of water and/or sugars and to the third overtone of carbonyl groups [26,28], shifted toward higher absorbance levels during fermentation due to the changes in water–casein micelle interactions and to the transformation of lactose into lactic acid [1]. Furthermore, protein structure changes during fermentation, including micelle rearrangement and interactions with acid, affected the N–H and C–H combination bands, related to the changes in the region 5600–6000 cm^−1^. In the region 5100–6000 cm^−1^, bacterial mass, pectin and cellulose seem also to be important. However, since water is a major component of yogurt, absorptions in this range were more likely attributed to the interactions between water and exopolysaccharides, which are produced by lactic acid bacteria and affect the yogurt texture [29].

However, scattering effects associated with changes in casein particle size [26] and in pH values of milk [29] induced baseline drift in the spectra, making it difficult to extract meaningful information from the raw spectral data. Furthermore, due to peak overlapping, the raw spectra exhibit poor spectral variations; thus, changes in chemical components are significantly masked by the strong absorbance bands of water. Therefore, appropriate spectral pre-processing is essential to enhance the spectral features associated with fermentation progression to highlight how changes in the dominant peaks are attributable to changes in water interactions with other compounds. Thus, standard normal variate (SNV) and first derivative transformation were applied, focusing only on the range 8900–5555 cm^–1^ (Figure 2B) to eliminate useless or saturated regions from the spectra [27,28]. After spectral transformation, the shift and reshape of water overtone and combination bands around 6900 cm^−1^, due to acid production and gelation, were more evident.

**Figure 3:**
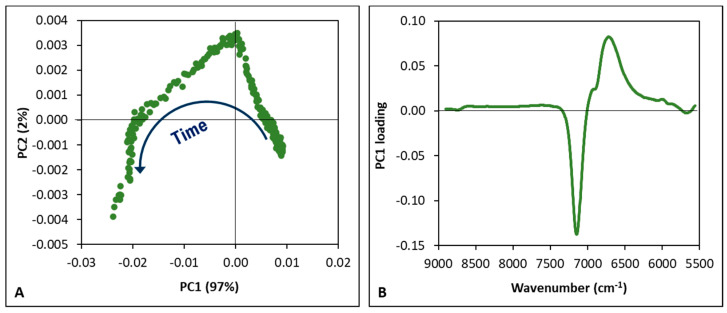
Principal Component Analysis of pre-processed FT-NIR spectra collected during one of the fermentation trials: (**A**) score plot; (**B**) loading plot.

The reduced and pre-processed spectra were then explored by PCA, separately for each fermentation trial, to uncover changes related to the time occurring during milk fermentation. Figure 3 shows an example of the results obtained for the purslane-fortified yogurt. In the PC1 vs. PC2 score plot (Figure 3A), a characteristic pattern of the spectra as a function of fermentation time is visible, similar to all the fermentation trials, with a total explained variance ranging from 98 to 100%. The PC1 loadings are visualized as spectral shape vectors (Figure 3B) to highlight the spectral feature information and enable spectral interpretation [35]. The plot highlights that the score pattern along the fermentation time was mainly influenced by the previously mentioned peak at 6900 cm^−1^, which, following the first derivative transformation, was deconvoluted into two distinct signals at 6700 and 7160 cm^−1^. Actually, as already mentioned, the main transformations occurring during lactic acid fermentation of milk are linked to a change in the interaction of casein micelles with water and to the transformation of lactose into lactic acid [1]. The high variance in these signals could be connected to the increase in hydrogen bonding and strongly bound water. Aquaphotomics investigations into the state of water within hydrogels have associated elevated absorbance in the spectral region around 6900 cm^−1^ with both the structural integrity of the hydrogel’s polymeric network and its capacity for water binding [29]. Thus, FT-NIR spectroscopy is able to capture both water–protein interactions and acidification phenomena, whereas pH measurement alone provides information only on the latter process.

**Figure 4:**
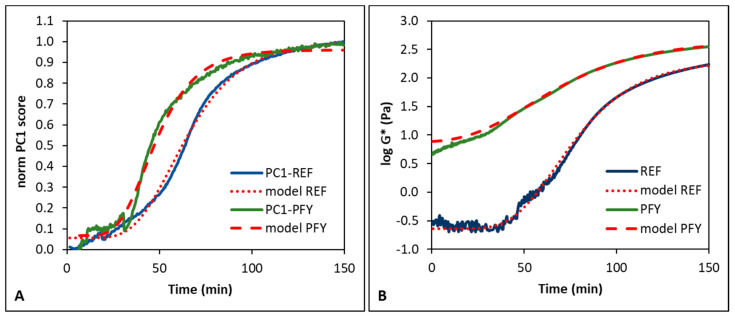
Average trends and Gompertz curves of normalized PC1 scores of pre-processed FT-NIR spectra (**A**) and complex modulus G* (**B**) as a function of fermentation time. REF, standard yogurt; PFY, purslane-fortified yogurt.

To have a clearer picture of the system trend during fermentation, the PC1 scores were normalized in the range 0–1 and plotted against the fermentation time (Figure 4A). The plot shows the characteristic sigmoidal trend typical of microbial metabolism, with an initial lag phase, followed by an exponential trend and a final stationary phase [36]. The shape of the fermentation curves depends on many experimental factors, such as the type of milk used, the presence of non-dairy ingredients, the starter culture and concentration, and the incubation temperature [24,36].

The curves were successfully described by the Gompertz equation (Figure 4A), with coefficients of determination higher than 0.977. The same equation also well-fitted the results obtained through the time sweep test (Figure 4B), with coefficients of determination higher than 0.921. In fact, the Gompertz equation is widely used to describe the kinetics of bioproduction processes. It can offer critical insights for elucidating and predicting the growth and production dynamics of a range of biological products. In particular, the Gompertz equation gives information about both the maximum growth rate and lag-time [36]. For instance, it was used by Soukoulis et al. [24] to describe the pH decrease and the apparent viscosity increase during milk fermentation for yogurt production, resulting in coefficients of determination higher than 0.938.

The kinetic critical points of the system evolution were extracted after the calculation of the first (d1) and second (d2) derivatives of the Gompertz curves (Table 2), as already performed in a previous work [28]. The critical points confirmed the faster development of the yogurt structure in the presence of the lyophilized purslane leaves. In fact, the acceleration (max d2), maximum rate (max d1), and deceleration (min d2) times were 10–68% shorter in PFY than in REF, considering both FT-NIR spectroscopy and rheological data. An early gelation was also observed with the addition of 1% freeze-dried apple pomace powder, which was attributed to depletion flocculation between the pectin in the apple pomace and caseins. This interaction led to the exclusion of polysaccharides around the casein micelles, resulting in stronger protein–protein interactions and aggregation at higher pH [8].

The results also showed that the FT-NIR spectroscopy was able to identify the critical points earlier than rheological measurements (i.e., times 18% earlier on average), as reported also by Grassi et al. [28]. Only the acceleration time of PFY was detected earlier when assessed using complex modulus data compared to spectral data. This discrepancy was attributed to the higher viscosity of the purslane-fortified milk, which hindered the detection of a distinct lag phase at the onset of fermentation (Figure 4B). The faster response of spectroscopy compared to rheology is explained by the ability of FT-NIR spectroscopy to detect not only physical changes but also chemical modifications in the systems, which are mainly associated with the first phases of milk coagulation [1]. Thus, the use of FT-NIR spectroscopy in process monitoring enables better control of the proper fermentation progression and allows for quicker intervention to address potential non-conformities. This is a very important result for food industries, which need fast, reliable, and simple methods for process control.

### 3.2. Yogurt Storage Monitoring

**Figure 5:**
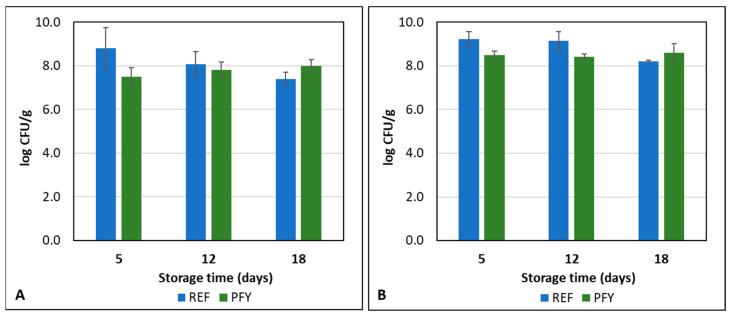
*Lb. bulgaricus* (**A**) and *Str. thermophilus* (**B**) concentrations in reference (REF) and purslane-fortified (PFY) yogurt samples during storage at 4 °C. Error bars represent the standard deviations of the two production replicates.

The microbial and textural properties of the yogurts were monitored during storage at 4 °C up to 18 days. Figure 5 shows the results of the lactic acid bacteria concentrations in the two yogurt types. No significant differences (*p* > 0.05) were found by considering both the yogurt type and the storage time. This means that the yogurt samples were stable during cold storage up to 18 days and the purslane addition did not affect microbial metabolism and survival. The sum of *Lb. bulgaricus* and *Str. thermophilus* was higher than 8.3 log CFU/g in all the samples, thus making the yogurts compliant with the Codex Alimentarius Standard indicating a minimum of 7 log CFU/g of starter microorganisms viable, active and abundant in the product to the date of minimum durability [37].

**Figure 6:**
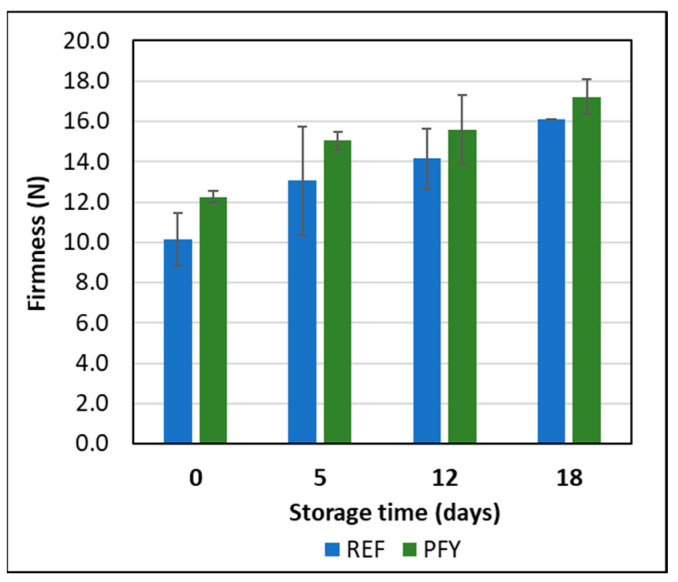
Firmness of reference (REF) and purslane-fortified (PFY) yogurt samples during storage at 4 °C. Error bars represent the standard deviations of the two production replicates.

For firmness (Figure 6), no significant differences (*p* > 0.05) were found between the two yogurt types nor the storage times, with the only exception of PFY at time 0, which was significantly (*p* < 0.05) less firm than the PFY samples at the other sampling times. No effects of purslane addition (0.5 and 1.0%) or storage time (up to 21 days at 4 ± 1 °C) on yogurt firmness were observed by other authors [12]. The slight increase in texture properties during storage was already observed in previous works as a consequence of the reinforcement of the gel structure at low temperature and the resulting higher water holding capacity [8,12,38]. The yogurt structural properties can be considered stable during storage and no syneresis was observed in the samples, with or without purslane addition.

## 4. Conclusions

In conclusion, this work demonstrated that it is possible to produce a purslane-fortified set-type yogurt without affecting its microbial or textural characteristics during both fermentation and storage. The presence of lyophilized purslane leaves made the milk more viscous, thus resulting in faster development of the gel structure. The development of its gel structure was assessed by both rheology and FT-NIR spectroscopy. Moreover, this work demonstrated that FT-NIR spectroscopy is an efficient tool for the in-line monitoring of the fermentation step, being able to highlight faster than rheology the kinetic critical points and thus providing the possibility of quickly intervening in cases of unconformities, allowing them to be corrected. This is extremely important for food industries looking for suitable analyzers to be used in the Process Analytical Technology (PAT) applied to process control in the frame of the Industry 4.0 program. In fact, NIR spectroscopy is one of the main e-sensing technologies used in PAT, being able to simultaneously analyze different food-related phenomena in a rapid, non-invasive and flexible way [39]. Moreover, NIR spectroscopy is by far more sustainable than wet chemistry, as recently demonstrated by Casson et al. [40].

Further studies should be conducted to evaluate consumers’ acceptability of the fortified yogurt, as well as possible changes during longer storage periods.

## Figures and Tables

**Table 1:** Concentrations (log CFU/g) of *Lb. bulgaricus* and *Str. thermophilus* in the reference (REF) and purslane-fortified (PFY) yogurt samples before (t0) and after (t2.5) fermentation.

Yogurt Type	*Lb. bulgaricus*		*Str. thermophilus*	
	**t0**	**t2.5**	**t0**	**t2.5**
REF	7.4 ± 1.0 ^a^	7.3 ± 0.1 ^a^	8.3 ± 1.1 ^a^	8.3 ± 0.1 ^b^
PFY	7.2 ± 1.1 ^a^	7.4 ± 0.4 ^a^	8.3 ± 1.0 ^a^	8.3 ± 0.1 ^a^

^a,b^, for the same yogurt type and sampling time, different superscript letters mean a significant difference (*p* < 0.05) in the concentration of *Lb. bulgaricus* and *Str. thermophilus*.

**Table 2:** Average kinetic critical points based on Gompertz curves of normalized PC1 scores of pre-processed FT-NIR spectra and logarithmic values of complex modulus (G*) for reference (REF) and purslane-fortified (PFY) yogurt samples.

Yogurt Type	Norm PC1 Curve			log G* Curve		
	Max d2 (min)	Max d1 (min)	Min d2 (min)	Max d2 (min)	Max d1 (min)	Min d2 (min)
REF	32.4	53.6	75.5	60.1	77.6	92.3
PFY	27.9	41.2	54.2	19.3	52.4	83.2

Max d2, acceleration time (corresponding to the maximum of the second derivative); Max d1, maximum rate time (corresponding to the maximum of the first derivative); Min d2, deceleration time (corresponding to the minimum of the second derivative).

## Data Availability

The data presented in this study are available on request from the corresponding author.
